# A new Korean Research Investment for Global Health Technology (RIGHT) Fund to advance innovative neglected-disease technologies

**DOI:** 10.1371/journal.pntd.0007956

**Published:** 2020-09-03

**Authors:** Peter J. Hotez, Kim Bush, Andrin Oswald, Glenn Rockman, In-taek Lim, Youngmee Jee, Chang Jin Moon, Jerome H. Kim, Younbeen Kim

**Affiliations:** 1 RIGHT Fund, Seoul, Republic of Korea; 2 Texas Children’s Hospital Center for Vaccine Development, National School of Tropical Medicine, Baylor College of Medicine, Houston, Texas, United States of America; 3 Life Sciences Partnerships, Bill & Melinda Gates Foundation, Seattle, Washington, United States of America; 4 Adjuvant Capital, New York, New York, United States of America; 5 Korean Ministry of Health and Welfare (MOHW), Sejong, Republic of Korea; 6 Center for Infectious Disease Research at the National Institute of Health, Korea Centers for Disease Control & Prevention (CDC), Osong, Republic of Korea; 7 International Vaccine Institute (IVI), Seoul, Republic of Korea; Yale University School of Medicine, UNITED STATES

## Abstract

In 2018, the government of the Republic of Korea (ROK), South Korean life science companies, and a group of international funders led by the Bill & Melinda Gates Foundation launched a new and innovative funding agency to support neglected-disease research and development (R&D). The new venture is known as the Research Investment for Global Health Technology (RIGHT) Fund.

## Introduction

At the end of the Korean War in 1953, ROK was a devastated nation and ranked among the poorest in the world. A decade later, in response to its high prevalence rates of poverty-related neglected diseases, including soil-transmitted helminth infections, malaria, and tuberculosis, the ROK government in collaboration with a Korean Association for Parasite Eradication (KAPE) began programs of infectious and parasitic disease control [1]. These activities, which included regular mass deworming of Korean schoolchildren between 1969 and 1995, together with economic development initiatives, dramatically reduced the prevalence of Korea’s neglected diseases [1, 2]. In some cases, KAPE and ROK government programs, together with steady economic growth, led to actual disease elimination [1, 2]. Moreover, because of the ability of neglected diseases to trap economies in poverty due to their chronic and debilitating effects on worker productivity and child development [3], ROK’s national successes in parasite control likely contributed to subsequent financial returns.

Today, the ROK boasts the 11th largest economy globally and one of the most advanced economies in Asia, with a gross domestic product (GDP) of 1.7 trillion USD [4]. Recognizing the historic impact of disease control in transforming its economy, beginning in the early 1990s the ROK government and its people advanced their own programs of official development assistance (ODA). The ROK ODA volume in 2017 was 2.64 trillion Korea won (2.64 billion USD), representing 0.16% of the gross national income (GNI), with a commitment to increase its ODA contribution to 0.3% of GNI by 2030.

## A new and innovative fund

A key element of the ROK’s economic success is its vigorous and productive life sciences and pharmaceutical industry, with advanced capabilities for drug, diagnostics, and vaccine development. The ROK has also well positioned to lead the convergence and integration of biotechnology, information and digital health, and communication technologies (ICT) for disease prevention, diagnosis, and treatment. These features afford a unique opportunity for the ROK to leverage and incorporate R&D into its programs of ODA.

Recognizing the ROK’s dual capacity to both contribute ODA at a high level and pursue a vigorous R&D agenda, the Bill & Melinda Gates Foundation pursued a partnership with both the ROK government and South Korea’s life sciences industry for developing an innovative fund for neglected-disease technologies. Such activities built on a previous success in Japan where the Global Health Innovative Technology (GHIT) Fund was established in 2013 [5]. However, the Korean initiative emphasizes the particular strengths of the South Korean life sciences and pharmaceutical industry and its major academic partners, together with the International Vaccine Institute (IVI) founded by the United Nations Development Programme in Seoul, ROK, in 1997.

**Fig 1:**
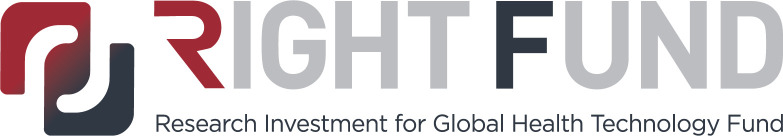
Logo of the new Korean RIGHT Fund. *From http://rightfund.org*. RIGHT, Research Investment for Global Health Technology.

In July of 2018, a new Research Investment for Global Health Technology (RIGHT) Fund was created as a three-way nonprofit partnership between the Ministry of Health and Welfare (MOHW) of the ROK government, South Korean life science companies, and the Gates Foundation, in addition to other international funders (Fig 1) [6].

Central to the RIGHT Fund is an innovative platform for the Korean partners to expand their contributions to global health and neglected-disease R&D projects, with a long-term objective to develop and deliver new neglected-disease drugs, diagnostics, and vaccines [6].

An overall project goal of the RIGHT Fund is “to advance the discovery and development of new health technologies to meet the needs of Low and Middle Income Countries (LMICs) by leveraging the intellectual, technological, and financial resources of the ROK” [6]. The Fund emphasizes new drugs, diagnostics, and vaccines, as well as new manufacturing technologies related to neglected diseases [6]. To date, it has mobilized more than 50 billion Korean won, or approximately 44 million USD, with approximately one-half of the contributions from the ROK government and the other 50% equally shared between Korean companies and the Gates Foundation.

## Structure and investment approaches

The RIGHT Fund operates through a management team that includes an Executive Director, together with individuals who lead operations in three areas—strategy, investment planning and management, and government relations. The RIGHT Fund management works with an independent selection committee and reports to a Board of Directors composed of representation from the Korean MOHW and Korean Centers for Disease Control & Prevention (CDC), in addition to an ex officio observer from the Gates Foundation, and members at large. Also overseeing the entire RIGHT Fund is a Council with representation from the Korean MOHW, National Institute of Health, IVI, Gates Foundation, and the leadership of the five major South Korean life sciences industries.

**Fig 2:**
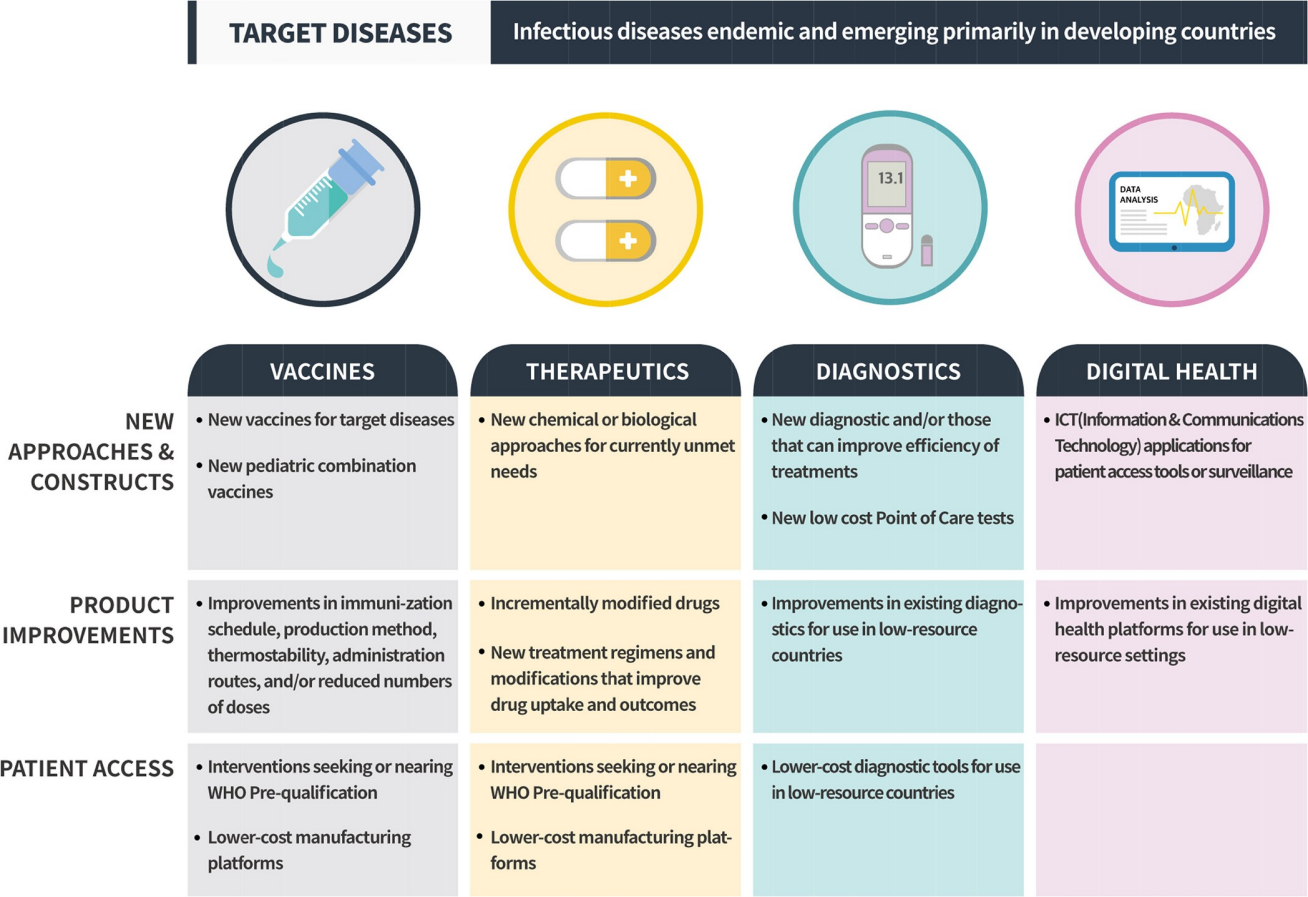
RIGHT Fund investment areas. *From http://rightfund.org*.

The technologies advanced by the RIGHT Fund are driven by the needs of the low-resource countries, typically measured through years of life lost or years lived with disability; their potential for impact; and whether they leverage the current strengths of life sciences R&D within ROK. An applicant should involve at least one Korean research organization or company. Another criterion is the potential of the technologies selected to expand the invovlement of Korean life sciencies organizations in the global health space and ecosystem. Fairness and transparency are also paramount. The three major technological areas include vaccines, therapeutic drugs, and diagnostics, with each area considering new approaches and constructs, product improvements, or patient access (Fig 2) [6]. Requests for proposals (RFPs) are issued periodically, and the proposals are evaluated for their eligibility and merit before the applicants are invited to submit full proposals. This process is followed by evaluation by the panel of experts, face-to-face interviews with the members of the selection committee, final selection, and issuance of a grant agreement with milestone-gated monitoring and disbursement of funds [6]. To avoid potential conflicts of interests, the RIGHT Fund has built a transparent governance structure in which there is a clear firewall between decision-makers and potential applicants. Moreover, the RIGHT Fund does not seek any profits or patent rights out of its funded projects but requires that all applicants accept its global access policies. This would ensure a commitment to make data and products resulting from funded projects available to their intended beneficiaries.

## New technologies

In its first year of operations, investments have been made in five technologies: two vaccines, one therapeutic agent, and two diagnostics. The two vaccines include (1) a novel conjugate cholera vaccine under joint development by Harvard Medical School, Massachusetts General Hospital, IVI, and EuBiologics (a Korean life sciences company) and (2) a hexavalent vaccine produced by LG Chem, another Korean life sciences company, which combines diphtheria whole-cell pertussis-tetanus toxoid vaccine, inactivated polio vaccine, recombinant hepatitis B vaccine, and *Haemophilus influenzae* type b (Hib) vaccine. The therapeutic is a low-cost manufacturing process for a new antimalaria drug from the product development partnership (PDP) Medicines for Malaria Ventures (MMV), together with the Korean life sciences company SK Biotek. The two diagnostics are (1) a second-generation test for glucose-6-phosphate dehydrogenase (G6PD) from the Seattle-based PDP, PATH, and the Korean life sciences company SD Biosensor and (2) a point-of-care diagnostic for detection of *Mycobacterium tuberculosis* and multidrug-resistance mutations from a PDP with diagnostics expertise, Foundation for Innovative New Diagnostics (FIND), and Bioneer, another Korean company [6]. The expectation is that this portfolio of new neglected-disease products will expand considerably in the coming years, in addition opening up the RIGHT Fund to mission critical coronavirus disease 19 (COVID-19) biotechnologies.

## Concluding comments

The concept of “blue marble health” finds an unexpected level of neglected-disease burden among the poor living in the Group of 20 (G20) nations [2]. Although poverty-related diseases—with a few exceptions, such as clonorchiasis and tuberculosis—are no longer endemic to the ROK, it has been further noted that the G20 also have a special obligation to support and advance the development of neglected-disease R&D [2]. Through the RIGHT Fund, the ROK government has made a major advance toward committing to investments in neglected-disease technologies and joins other important partner countries, including the United States, United Kingdom, and Japan, on this front. In anticipation of downstream successes, the Korean RIGHT Fund could serve as a template for similar neglected-disease technology partnerships from other key G20 nations.
